# Early Normalization of Squamous Cell Carcinoma Antigen During Combined Chemoradiation Predicts Pathological Response and Survival in Squamous Cervical Cancer: A Retrospective Cohort Study

**DOI:** 10.3390/cancers18142225

**Published:** 2026-07-10

**Authors:** Christoph Ebner, Linda Ebner, Sergej Skvortsov, Heidelinde Fiegl, Katharina Steger, Barin Feroz, Verena Wieser, Katharina Leitner, Irina Tsibulak, Christian Marth, Alain Gustave Zeimet

**Affiliations:** 1 Department of Obstetrics and Gynecology, Medical University Innsbruck, 6020 Innsbruck, Austria; christoph.ebner@i-med.ac.at (C.E.); linda.ebner@i-med.ac.at (L.E.); heidelinde.fiegl@i-med.ac.at (H.F.); katharina.steger@i-med.ac.at (K.S.); barin.feroz@i-med.ac.at (B.F.); verena.wieser@i-med.ac.at (V.W.); katharina.leitner@i-med.ac.at (K.L.); irina.tsibulak@i-med.ac.at (I.T.); christian.marth@i-med.ac.at (C.M.); 2 Department of Therapeutic Radiology and Oncology, Medical University of Innsbruck, 6020 Innsbruck, Austria; sergej.skvortsov@i-med.ac.at

**Keywords:** squamous cell carcinoma antigen, SCC-A, cervical cancer, chemoradiation, biomarker, prognosis

## Abstract

**Objective:** Squamous cell carcinoma antigen (SCC-A) is a widely used biomarker for squamous cell cervical carcinoma and pretreatment elevation is associated with poor prognosis. Normalization during chemoradiation correlates with PET-CT response and survival. This study assessed the prognostic value of SCC-A normalization for biopsy-proven pathological response and survival outcomes. **Materials and Methods:** This retrospective single-center cohort study included patients with locally advanced or node-positive squamous cell cervical cancer treated with definitive chemoradiation at the Medical University Innsbruck between 2008 and 2023. Eligible patients had baseline SCC-A ≥ 2 ng/mL and at least two additional measurements within 42 days of treatment. SCC-A normalization was evaluated at predefined weekly time points. Associations with biopsy-assessed residual disease, PFS, and OS were assessed. **Results:** Of 186 screened patients, 83 met the inclusion criteria. Within 42 days, 70% achieved SCC-A normalization, with a median time of 21 days (IQR 19–32). Among predefined time points, normalization by day 28 was associated with reduced odds of residual disease (OR 0.14; 95% CI 0.04–0.44) and improved PFS (HR 0.28; 95% CI 0.12–0.63) and OS (HR 0.37; 95% CI 0.14–0.96), remaining independently significant after multivariate adjustments. **Conclusions:** SCC-A normalization during chemoradiation is a non-invasive independent biomarker of treatment response. Normalization within 28 days identifies patients at low risk of residual disease, progression, and death, supporting its use for early risk stratification and response monitoring for potential treatment adaptations.

## Simple Summary

Squamous cell carcinoma antigen (SCC-A) is a well-established tumor marker for cervical cancer. Elevated pretreatment levels are associated with larger tumors, lymph node involvement, and worse survival. While SCC-A is routinely measured before and after treatment, the prognostic value of its dynamics during chemoradiation has rarely been studied. In this retrospective study of 83 patients with squamous cell cervical cancer, we investigated whether the speed of SCC-A decline during chemoradiation carries prognostic information. We show that patients who achieved SCC-A normalization within 28 days of treatment initiation had significantly lower odds of biopsy-proven residual disease at the end of treatment, and were at significantly lower risk of disease progression and death. These associations remained independently significant after adjustment for established prognostic factors. Our findings suggest that monitoring SCC-A kinetics during chemoradiation is a simple, non-invasive, and widely available strategy for early risk stratification that may help guide individualized treatment decisions.

## 1. Introduction

Cervical cancer is the fourth most common cancer in women worldwide, with an estimated 662,301 new cases and 348,874 deaths in 2022 [1]. Nearly all cases are attributable to persistent infection with high-risk human papillomavirus [2]. Squamous cell carcinoma antigen (SCC-A) is a well-established tumor marker for cervical squamous cell cancer. Elevated pretreatment serum levels are associated with larger tumor size, regional lymph node involvement, higher risk of recurrence and higher mortality. Beyond cervical cancer, SCC-A is an established serum tumor marker for squamous cell carcinomas including the head and neck or lung malignancies, where elevated levels are similarly associated with tumor burden and adverse prognosis [3,4,5]. While most often only pretreatment and post-treatment SCC-A measurements are obtained, there is evidence that tumor marker dynamics during chemoradiation is of predictive (with regard to metabolic positron emission tomography–computed tomography (PET-CT) response) and prognostic value [6,7]. Serial SCC-A monitoring after treatment can also detect recurrence with a lead time of several months before clinical manifestation [8], further supporting its role in disease surveillance.

Biologically, SCC-A is not merely a passive tumor marker but a functional serine protease inhibitor. Clinical SCC-A assays measure the two homologous isoforms SERPINB3 (SCC-A1) and SERPINB4 (SCC-A2), which are physiologically expressed by squamous epithelia and are involved in the regulation of apoptosis and cell survival. SERPINB3 has been shown to protect cervical cancer cells from radiation-induced cell death through inhibition of the lysosomal cysteine protease cathepsin L, and its genetic knockout increases the radiosensitivity of cervical tumor cell lines. This mechanistic link provides a rationale for why the dynamic behavior of SCC-A during treatment, rather than a single static measurement, may reflect the intrinsic radiation sensitivity of the tumor and thereby carry prognostic information [9].

Definitive chemoradiation with concomitant cisplatin followed by MRI-guided adaptive brachytherapy is the standard of care for locally advanced cervical cancer and has substantially improved local control [10]; nevertheless, a substantial proportion of patients still experience locoregional or distant treatment failure [11,12]. The optimal method and timing of response evaluation after combined chemoradiation remain a matter of discussion. Available methods include magnetic resonance imaging (MRI), PET-CT, ultrasound, or less commonly tissue biopsy. Both radiological and histological assessments are usually performed weeks or even months after completion of chemoradiation, due to delayed radiation effects that may lead to continued tumor response even after the last radiation dose has been applied [13,14].

At the University Hospital Innsbruck, we traditionally perform routine biopsies of the tumor bed or, if visible, the residual tumor at the time of the last brachytherapy. If persistent cervical cancer is detected, biopsies are repeated 6 weeks later. As recently reported, patients with residual cancer at the time of the last brachytherapy experience significantly worse progression-free survival (PFS) even if complete pathological response is detected later at the secondary biopsy. These findings suggest that lack of early response constitutes a reliable marker of poor outcome, which appears to be impactful only at the end of radiotherapy [15]. However, ESGO/ESTRO/ESP guidelines discourage routine biopsies by the fear that performing biopsies in irradiated tissue may increase the risk of fistula development [16,17].

The concept of deriving prognostic information from the kinetics of a serum tumor marker during treatment is well-established in other gynecological malignancies. In epithelial ovarian cancer, the modeled CA-125 elimination rate constant K (KELIM), derived from serial CA-125 measurements during chemotherapy, has emerged as a reproducible indicator of intrinsic tumor chemosensitivity. In a recent systematic review and meta-analysis comprising 14,444 patients, a favorable KELIM score was associated with significantly improved progression-free survival (HR 0.53, 95% CI 0.45–0.62) and overall survival (HR 0.51, 95% CI 0.43–0.62) in primary ovarian cancer and was found to outrank conventional single-timepoint response criteria. This demonstrates that modeling the longitudinal decline of a marker can provide prognostic information beyond that of static measurements. Whether an analogous kinetic principle applies to SCC-A during chemoradiation for cervical cancer has not been systematically investigated, which is addressed in the present study by examining the timing of SCC-A normalization as an early prognostic parameter [18,19].

The relevance of early response assessment during primary treatment is increasing due to options of maintenance therapy as well as new drugs that have been investigated within the last decade and which could be particularly beneficial in early non-responders. Furthermore, early prognostic biomarkers may decisively support an additional high-ranking challenge in precision medicine, which is the fast and reliable identification of patients, who can be spared from such treatments because of their excellent prognosis. One should not disregard that even though extension of combined chemoradiation by immunotherapy and/or anti-angiogenic therapy may show benefit in a large number of patients in cervical cancer [20,21,22,23], these treatments may be associated with a substantial risk of severe toxicity in some patients [24]. Of note, immunotherapy in the locally advanced setting remains an expensive treatment option and is currently not considered cost-effective [25].

Acknowledging the potential risks of routine biopsies, this study based on SCC-A dynamics aims to establish a non-invasive valuable alternative to predict the pathological response and survival outcome and may therefore guide escalation or de-escalation of adjuvant treatment.

## 2. Materials and Methods

### 2.1. Study Design and Patient Data

This was a retrospective single-center observational cohort study including all patients undergoing combined chemoradiation for locally advanced or node-positive squamous cell cervical cancer treated at the Department of Obstetrics and Gynecology and at the Department of Therapeutic Radiology and Oncology, Medical University of Innsbruck, Innsbruck, Austria, between 2008 and 2023. Patients with squamous cell carcinoma of the uterine cervix were identified from the tumor registry of the Department of Gynecology and Obstetrics, Medical University of Innsbruck, based on diagnosis and tumor stage. Patient and treatment data was extracted from the digital medical records. All of the identified patients, who underwent combined chemoradiation for locally advanced or node-positive cervical cancer with curative intent, were included in this study provided their baseline serum SCC-A was equal to or exceeded the routine cutoff level between normal and pathologic of 2 ng/mL at the start of combined chemoradiation and at least two additional SCC-A values were documented within the first six weeks after the start of chemoradiation therapy. Patients with neoadjuvant chemotherapy, palliative treatment intent, and SCC-A < 2 ng/mL at baseline or non-squamous histology were excluded. Staging was performed according to FIGO 2018 criteria for all patients. Tumors which were originally staged according to FIGO 2009 were retrospectively converted to FIGO 2018 for this study. Nodal staging was performed either surgically by laparoscopic sampling or PET-CT.

### 2.2. Treatment and SCC-A Measurement

Patients received definitive external beam radiotherapy (EBRT) using three-dimensional conformal radiotherapy (3D-CRT) or volumetric modulated arc therapy (VMAT) on Elekta (Elekta AB, Stockholm, Sweden) linear accelerators, combined with weekly platinum-based chemotherapy (cisplatin 40 mg/m^2^). EBRT was delivered in daily fractions of 1.8 Gy to a median total dose of 45–50.4 Gy to the pelvis with or without para-aortic lymph nodes, including nodal boosts up to 60 Gy (EQD2), with a transition from 3D-CRT with central shielding before 2017 to predominantly VMAT or intensity-modulated radiotherapy thereafter. Brachytherapy was performed using an Elekta Flexitron^®^ (Elekta AB, Stockholm, Sweden) afterloading system, evolving from weekly intracavitary applications (5 × 7 Gy) during EBRT to MRI-guided adaptive intracavitary/interstitial brachytherapy (4 × 7 Gy) after completion of EBRT from 2017 onward, in accordance with the EMBRACE-II protocol [26]. The technical evolution of definitive radiotherapy at our institution over the study period has been described in detail previously [27]. SCC-A was assessed during routine blood work prior to each administration of cisplatin as well as once a week during brachytherapy. Serum levels of the tumor marker were measured using chemiluminescence microparticle immunoassay on an Architect i1000SR immunoassay analyzer (Abbott Diagnostics, Abbott Park, IL, USA). As a routine response evaluation after combined chemoradiation at the Medical University of Innsbruck, systematic biopsies of the cervix or the tumor bed or macroscopically visible tumor were obtained at the time of the last brachytherapy. At least four histologic specimens were obtained per patient. If a macroscopic, clearly recognizable residual tumor was present, a punch biopsy was obtained from this region, complemented by random punch biopsies at the 12, 3, 6, and 9 o’clock positions together with an endocervical curettage. In the case of a complete macroscopic response, only the four random biopsies of the tumor bed and the curettage were performed. These biopsies are part of our standard routine and are performed on every patient regardless of macroscopic response. The exact method for obtaining the biopsies as well as their prognostic value was recently described [15].

### 2.3. Statistics

The primary endpoints of this study were pathologic complete response at the end of brachytherapy, progression-free survival and overall survival. Pathologic complete response was defined as the absence of tumor cells in biopsies obtained at the time of last brachytherapy.

Continuous variables are reported as medians with interquartile ranges (IQRs), and categorical variables as counts and percentages. Baseline was defined as the serum SCC-A value measured closest to the start of radiotherapy. If two measurements were equally distant from radiotherapy start, the earlier value was selected. Post-treatment SCC-A kinetics are summarized using patient-level weekly aggregates (median per patient-week). SCC-A normalization was defined as the first value < 2 ng/mL after radiotherapy start within prespecified time frames. To identify the earliest tumor marker reversal point with clinically relevant prognostic value, SCC-A normalization was evaluated at predefined time points (7, 14, 21, 28, 35, and 42 days after treatment initiation), with patients divided at each time point into those with and without normalization and compared with respect to pathological response and survival outcomes. Very early and very late thresholds were expected to yield only poor statistical power because of small and/or highly imbalanced group sizes; therefore, group sizes and event counts were explored for each weekly evaluation. In addition to the predefined time points, an exploratory data-driven optimal time point at which SCC-A normalization yielded the highest prognostic value was identified for all endpoints using a day-wise approach (days 1–56 after treatment initiation). For pathologic response, the optimal time point was defined as the day with the lowest *p*-value derived from Fisher’s exact test. For progression-free survival and overall survival, univariable Cox proportional hazards models were fitted for each day, and the optimal time point was defined as the day with the lowest *p*-value based on the Cox score test.

Associations between early tumor marker normalization and complete pathological response at the end of brachytherapy were assessed using logistic regression, with results reported as odds ratios and 95% confidence intervals. Survival outcomes were defined as the time from primary diagnosis to first detection of progression or death for progression-free survival or death for overall survival. Administrative censoring was applied at 60 months, reflecting the end of routine in-hospital follow-ups at our institution. Survival distributions were estimated using the Kaplan–Meier method and compared using the log-rank test. The Cox proportional hazards model was used to calculate hazard ratios, and for univariable Cox models, *p*-values were derived from the score test, whereas *p*-values in multivariable Cox models were based on the Wald-test. For all analysis, the cohort was split in an early versus not-early SCC-A normalization group at the respective predefined evaluation days. Multivariable Cox regression was used to adjust for prespecified confounders, including FIGO stage (≤II vs. ≥III), lymph-node status (positive vs. negative), and baseline SCC-A modeled on the log2 scale (hazard ratios per doubling). Multivariate analysis was performed on a complete case basis for all covariates. All analyses were conducted using R (version 4.5.2; R Foundation for Statistical Computing, Vienna, Austria).

## 3. Results

**Figure 1:**
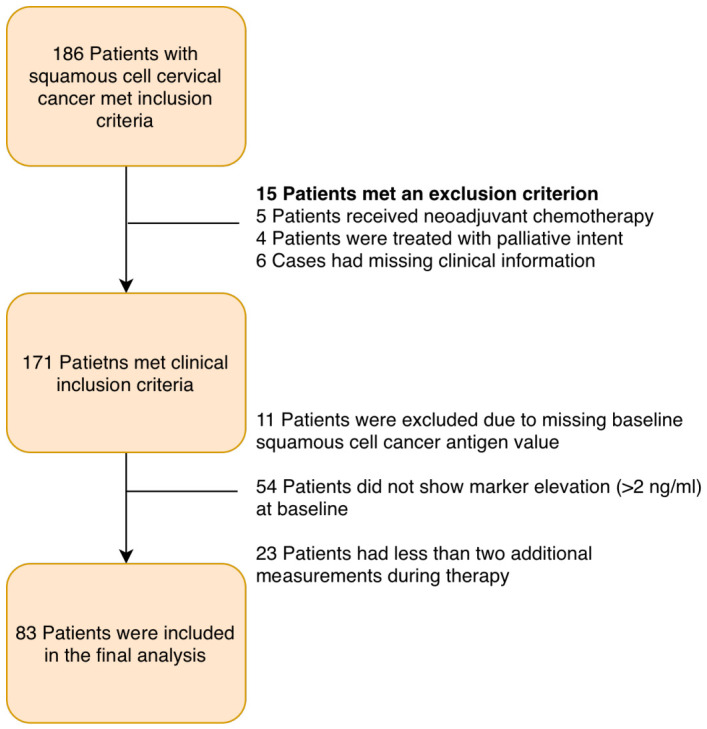
Patient selection flowchart showing the inclusion and exclusion of patients with squamous cell cervical cancer.

A total of 186 patients with squamous cell cervical cancer met inclusion criteria. Of these patients, 15 were excluded due to prespecified clinical exclusion criteria or missing clinical information, 11 patients had no available SCC-A measurement at baseline and 54 had a baseline measurement below the prespecified cutoff of 2 ng/mL. Finally, 23 patients had fewer than two additional tumor marker measurements during the 6 weeks after the first radiotherapy, thus leaving 83 patients for further analysis (Figure 1).

The median number of recorded SCC-A measurements in the timespan of 6 weeks after the start of radiotherapy per patient was 4 (IQR 3–5). The median interval between measurements was 7 days (IQR 7–8). Clinical and disease characteristics of this cohort are described in Table 1.

**Figure 2:**
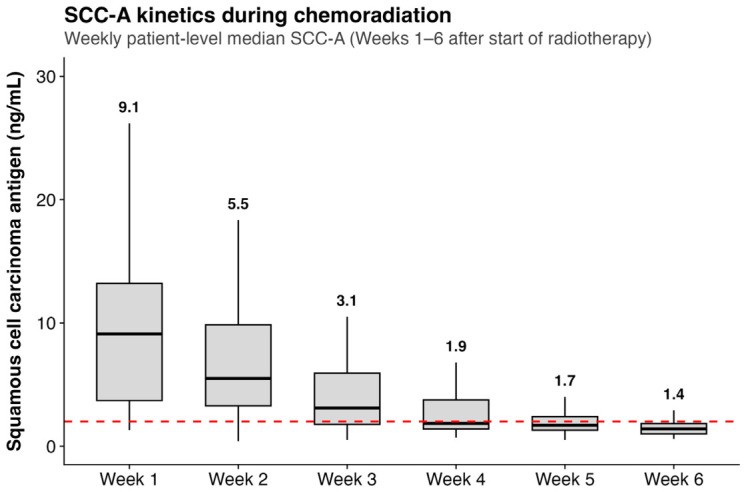
SCC-A kinetics during chemoradiation. Boxplots show weekly patient-level median SCC-A concentrations (ng/mL) from weeks 1–6 after treatment start. Numbers above boxes indicate cohort medians. The dashed red line marks normalization (2 ng/mL). Weeks correspond to days 1–7, 8–14, 15–21, 22–28, 29–35, and 36–42 after initiation.

The median SCC-A value at baseline was 7.6 ng/mL (IQR 4.1–14) and 9.7 ng/mL after the first week of radiotherapy. Within the 6-week interval (0–42 days), 70% of patients (58/83) achieved SCC-A normalization (value < 2 ng/mL). The median time to normalization was 21 days (IQR 19–32, mean ± SD: 25 ± 9.1 days) corresponding to the last day of therapy week 3 (Figure 2).

### 3.1. Prediction of Biopsy-Assessed Pathological Complete Response

**Figure 3:**
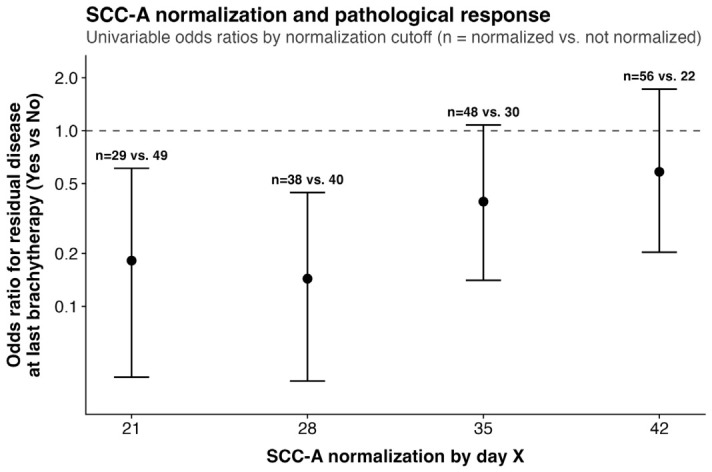
Association between SCC-A normalization and pathological response at last brachytherapy. Univariable logistic regression at days 21, 28, 35, and 42 from radiotherapy start shows odds ratios for residual disease in patients with versus without SCC-A normalization. Numbers indicate patients with normalized and non-normalized SCC-A at each cutoff timepoint.

Information on pathological response at the time of the last brachytherapy was available for 78 of the 83 included patients (94%). Of these, 56 patients (71.8%) showed no residual disease, whereas 22 patients (28.2%) had histologically detectable residual cancer at the end of combined chemoradiation. Measured SCC-A normalization early during chemoradiation was associated with significantly reduced odds of residual disease at the end of treatment. Among the predefined cutoffs, the strongest association was observed for normalization by day 28 (odds ratio 0.14, 95% confidence interval 0.04–0.44; *p* = 0.002). On day 28, SCC-A normalization yielded a negative predictive value for a pathological complete response of 90%, with a sensitivity of 82%, a specificity of 61%, and a positive predictive value of 45%. At later cutoffs, the association attenuated and was no longer statistically significant (35 days: odds ratio 0.39, *p* = 0.071; 42 days: odds ratio 0.58, *p* = 0.318) (Figure 3).

A data-driven day-by-day analysis identified day 24 as the optimal time point for SCC-A normalization with respect to pathological complete response. By day 24, 35 patients had normalized tumor marker levels, whereas 43 patients had persistently elevated levels. SCC-A normalization by day 24 was associated with lower odds of biopsy-proven residual disease at the time of the last brachytherapy (odds ratio 0.12, 95% confidence interval 0.02–0.48; *p* < 0.001). The probability of a pathological complete response given normalization by day 24 was 91.4% (32/35).

### 3.2. Recurrence Patterns According to Timepoint of SCC-A Normalization

Among the 83 patients included in this study, 24 (28.9%) experienced disease recurrence during follow-up. Of these, 5 patients (20.8%) had a local recurrence, and 19 patients (79.2%) developed distant metastases. Evaluation of recurrence patterns regarding normalization status on day 28 showed that none of the five patients with local recurrence achieved SCC-A normalization by day 28, and among patients with distant recurrence, only 3 of 19 (15.8%) normalized.

### 3.3. Survival Outcomes

**Figure 4:**
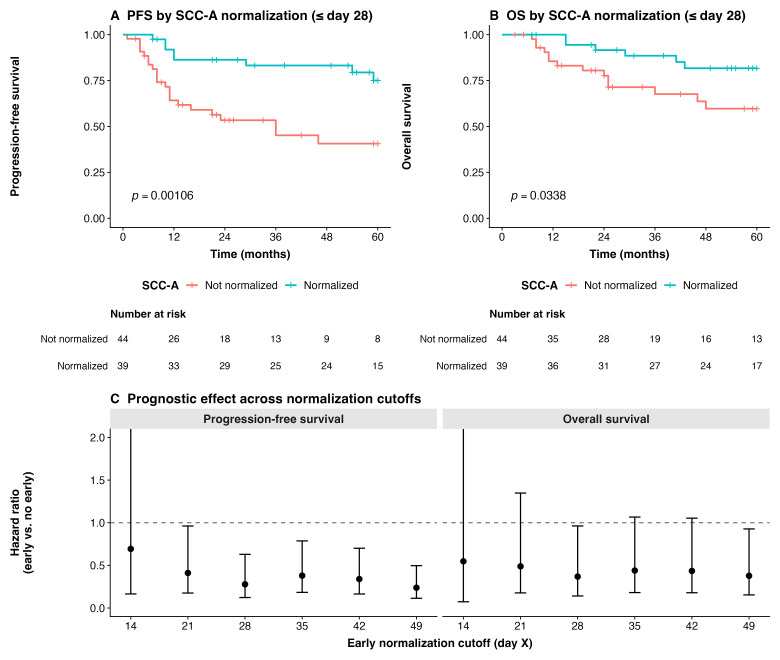
(**A**,**B**) Kaplan–Meier curves for progression-free survival and overall survival stratified by SCC-A normalization within 28 days after start of radiotherapy (SCC-A < 2 ng/mL). (**C**) Hazard ratios for early versus no early SCC-A normalization across weekly timepoints.

The median follow-up was 60 months for overall survival and 59 months for progression-free survival, calculated using the reverse Kaplan–Meier method with administrative censoring at 60 months. Patients with a normalized SCC-A by day 28 after the start of chemoradiation showed a significantly improved progression-free survival (HR 0.28, 95% CI 0.12–0.63, *p* = 0.0011) and overall survival (HR 0.37, 95% CI 0.14–0.96, *p* = 0.034). Analysis at both very early and very late timepoints yielded reduced statistical efficiency due to small and highly imbalanced group sizes. Group sizes were best balanced when dichotomization of the study cohort was made on day 28, with 39 patients with normalized SCC-A vs. 44 with a serum value still above the cutoff of 2 ng/mL (Figure 4).

After adjustment for baseline SCC-A level (log_2_), FIGO stage (≤II vs. ≥III), and lymph node involvement, early SCC-A normalization within 28 days remained independently associated with improved progression-free survival (HR 0.13, 95% CI 0.04–0.37; *p* < 0.001) and overall survival (HR 0.22, 95% CI 0.06–0.76; *p* = 0.017) in the multivariable Cox regression analysis.

In a day-by-day analysis the strongest prognostic effect for progression-free survival was observed for SCC-A normalization by day 48, which was associated with a significantly reduced risk of disease progression (HR 0.24, 95% CI 0.11–0.50; *p* < 0.001). For overall survival, the optimal time point was identified on day 50, with early SCC-A normalization also being associated with significantly improved survival (HR 0.33, 95% CI 0.13–0.80; *p* = 0.010).

### 3.4. Additional Exploratory Analyses

Among the 54 patients excluded because of a baseline SCC-A below 2 ng/mL, clinical follow-up data were available for 48. In an exploratory analysis, these SCC-A-negative patients showed progression-free and overall survival comparable to patients who achieved early normalization (PFS *p* = 0.42, OS *p* = 0.73) and a significantly better prognosis than patients without early normalization (PFS *p* = 0.004, OS *p* = 0.045).

Salvage hysterectomy after combined chemoradiation was performed in only five patients in our cohort. Two of these patients had achieved early SCC-A normalization by day 28, whereas three had not.

## 4. Discussion

To our knowledge this is the first study to show that biopsy-proven histological response in cervical squamous cell cancer can be predicted using a serological marker. Failure of normalization within the first weeks of chemoradiation identifies a group of patients that are at higher risk of residual disease at the end of combined chemoradiation as well as disease progression and death.

Here, we demonstrate that early SCC-A normalization by day 24 can predict pathological response with a high negative predictive value of 91.4%. SCC-A normalization at timepoints later than 28 days could not reliably predict pathological response. Concerning survival, the day-by-day analysis identified day 48 for progression-free survival (HR 0.24, 95% CI 0.11–0.50) and day 50 for overall survival (HR 0.33, 95% CI 0.13–0.80) as numerically optimal; however, the predefined time point on day 28 showed clinically comparable effect sizes (progression-free survival: HR 0.28, 95% CI 0.12–0.63; overall survival: HR 0.37, 95% CI 0.14–0.96) while also providing a robust prediction of biopsy-assessed residual disease (OR 0.14, 95% CI 0.04–0.44; *p* = 0.002). Day 28 appears particularly suitable for clinical implementation, as it occurs at a time within the treatment plan that still allows potential adaptation of subsequent management. Failure to achieve early SCC-A normalization was observed across both local and distant recurrences, suggesting that early SCC-A kinetics reflect overall treatment response rather than a specific pattern of failure.

The majority of published literature on the prognostic implication of SCC-A is focused on the initial elevation before treatment or on the dynamics between baseline and the time after completion of chemoradiation [3,7,28,29]. Markovina et al. were the first to demonstrate a correlation between SCC-A dynamics during radiotherapy and metabolic response evaluated by PET-CT approximately three months later. The findings of the present study are in agreement with their reported findings on survival outcomes, with SCC-A normalization around day 28 (comparable to day 27 in their cohort) being significantly associated with improved disease-free survival and overall survival. Of special note is that our approach provides the first evidence linking early SCC-A normalization to biopsy-proven pathological response [6].

Recently, tumor-informed ultrasensitive ctDNA analyses from the phase III CALLA trial demonstrated that persistent ctDNA detection after chemoradiation is strongly associated with relapse and inferior survival [30]. These findings strongly support a biomarker-based approach to monitor treatment response. Nonetheless, monitoring tumor-informed ctDNA requires individualized genomic profiling and specialized sequencing infrastructure, whereas SCC-A testing is widely available and inexpensive and may therefore constitute a pragmatic alternative for early risk stratification in routine clinical practice.

The main limitations of this study are its retrospective single-center design and the limited sample size. However, expansion to a retrospective multicenter cohort is constrained by the requirement for high SCC-A measurement density during chemoradiation, which is not routinely achieved in most institutions. As reported, 54 of 171 patients (31.6%) had to be excluded because of a pretreatment SCC-A concentration below the threshold of 2 ng/mL, implying that these cases are unable to benefit from this approach based on an SCC-A normalization for risk stratification.

By nature of the study design our results may be affected by limitations inherent to retrospective cohort studies, including selection bias, residual confounding, and heterogeneity in follow-up and management strategies over the study period. We carefully sought to minimize potential sources of bias by applying strict inclusion criteria and predefined clinically meaningful weekly time-points at which normalization vs. non-normalization was evaluated. The additional calculation of a data-driven ideal cutoff day should be regarded as exploratory. Given the limited sample size relative to the number of covariates included in the multivariable model, overfitting cannot be excluded, and the multivariable results should be interpreted with caution. We attempted to expand the cohort through multicenter collaborations; however, as serial SCC-A measurement during chemoradiation is not standard clinical practice in other institutions, no partner with adequate measurement density could be recruited.

We are aware that the requirement for at least two additional SCC-A measurements may have preferentially selected patients with better follow-up. In an exploratory comparison, excluded patients differed only in older age (median, 69.5 versus 54.0 years) but not in FIGO stage, lymph node status, or survival. HPV status, performance status, comorbidities, and molecular characteristics were not systematically recorded across the study period and may represent residual confounders. The possibility of lead-time bias cannot be excluded, as early SCC-A normalization may reflect inherently favorable tumor biology rather than a modifiable treatment response, and a causal relationship cannot be established from this retrospective analysis. Although day 28 was one of the predefined weekly time points, its selection as the primary cutoff was informed by this cohort and requires external validation.

Notably, this cohort was treated before the publication of the KEYNOTE-A18 trial and the resulting introduction of immunotherapy as routine maintenance, allowing assessment of SCC-A normalization as an independent prognostic marker without being confounded by immune checkpoint inhibition.

Routine consecutive SCC-A measurements are a safe and relatively inexpensive strategy for treatment monitoring. From a patient perspective, no additional blood draws are required, since SCC-A can be assessed using blood samples collected during weekly cisplatin administration. Based on data from this cohort, measurements at baseline, at around day 24 as well as around day 50 of therapy, would yield meaningful prognostic insights and could be implemented in clinical routine without much effort.

The use of SCC-A normalization as an early prognostic marker may support a more individualized approach to maintenance therapy, helping to distinguish patients who may benefit most from immune- and/or anti-angiogenic therapy from those with an inherently favorable prognosis and potentially sparing them unnecessary toxicity while reducing overall treatment costs.

Patients with persistent cervical cancer after conclusion of chemoradiation can benefit from salvage surgery as part of an individualized treatment plan. When surgery is a valid option, early detection of persistent disease has been shown to be superior compared to a later identification, as diagnosis within 62 days after completion of radiotherapy was associated with significantly improved progression-free and overall survival, as well as higher rates of complete resection with tumor-free margins [31]. Our data complement these findings by providing a biological risk stratification, allowing identification of a high-risk subgroup in whom intensified monitoring and earlier biopsy to detect persistent disease should be considered so that potentially indicated salvage surgery can be performed within this 62-day window.

Large prospective clinical studies with standardized SCC-A sampling schedules as part of the therapy monitoring are warranted to validate the present findings and, more importantly, to prove a potential clinical utility of early SCC-A-guided risk stratification. Respective trials would be especially crucial when they focus on the identification of potential candidates for salvage surgery or on the evaluation of early treatment switches or indications for maintenance therapies and their eventual prolongations.

## 5. Conclusions

Serial SCC-A measurements during chemoradiation represent a clinically meaningful tool for risk stratification in patients with locally advanced cervical cancer. By enabling early identification of patients at increased risk for persistent disease, SCC-A kinetics have the potential to inform individualized adaptive treatment strategies, including timely consideration of salvage approaches. Given that SCC-A measurement is non-invasive, safe, inexpensive, and easy to combine with routine blood work during chemotherapy administration, its integration into routine clinical workflows is feasible.

## Figures and Tables

**Table 1:** Baseline characteristics and SCC-A measurement density of the study cohort (*n* = 83).

**Variable**	**Overall (** ***n*** **= 83)**	**No Normalization ≤ Day 28 (n = 44)**	**Normalization ≤ Day 28 (n = 39)**
Age at diagnosis, median [IQR]	54.0 [44.0–63.5]	55.5 [47.8–61.3]	53.5 [42.0–64.0]
FIGO stage, *n* (%)			
FIGO I	4 (4.8)	2 (4.5)	2 (5.1)
FIGO II	18 (21.7)	7 (15.9)	11 (28.2)
FIGO III	41 (49.4)	19 (43.2)	22 (56.4)
FIGO IV	10 (12.0)	8 (18.2)	2 (5.1)
Unknown	10 (12.0)	8 (18.2)	2 (5.1)
Tumor grading, *n* (%)			
G1	1 (1.2)	1 (2.3)	0 (0.0)
G2	30 (36.1)	16 (36.4)	14 (35.9)
G3	37 (44.6)	19 (43.2)	18 (46.2)
Unknown	15 (18.1)	8 (18.2)	7 (17.9)
Lymph node positive, *n* (%)			
No	20 (29.9)	10 (29.4)	10 (30.3)
Yes	47 (70.1)	24 (70.6)	23 (69.7)
EBRT technique, *n* (%)			
3D-CRT	37 (47.4)	21 (52.5)	16 (42.1)
VMAT/IMRT	41 (52.6)	19 (47.5)	22 (57.9)
HRCTV, median [IQR]	39.9 [27.6–56.6]	43.2 [28.8–68.7]	38.9 [26.8–47.5]
D90HRTV, median [IQR]	84.8 [81.6–87.2]	83.0 [77.1–86.5]	85.2 [83.7–88.9]
Baseline SCC-A, median [IQR]	7.6 [4.1–14.2]	9.2 [5.4–23.8]	6.3 [2.9–11.2]
Chemotherapy cycles, *n* (%)			
<4	9 (10.8)	7 (15.9)	2 (5.1)
≥4	74 (89.2)	37 (84.1)	37 (94.9)

## Data Availability

The datasets generated and/or analysed during the current study are not publicly available due to institutional data protection regulations but are available from the corresponding author upon reasonable request.

## Abbreviations

The following abbreviations are used in this manuscript:

SCC-A	Squamous cell carcinoma antigen
PFS	Progression-free survival
OS	Overall survival
FIGO	International Federation of Gynecology and Obstetrics
EBRT	External beam radiotherapy
3D-CRT	Three-dimensional conformal radiotherapy
VMAT	Volumetric modulated arc therapy
IMRT	Intensity-modulated radiotherapy
IQR	Interquartile range
HR	Hazard ratio
OR	Odds ratio
CI	Confidence interval
MRI	Magnetic resonance imaging
PET-CT	Positron emission tomography–computed tomography
ctDNA	Circulating tumor DNA
NPV	Negative predictive value
PPV	Positive predictive value
EQD2	Equivalent dose in 2 Gy fractions
